# Biochemical and nutritional characterization of the medfly gut symbiont *Enterobacter* sp. AA26 for its use as probiotics in sterile insect technique applications

**DOI:** 10.1186/s12896-019-0584-9

**Published:** 2019-12-18

**Authors:** Konstantinos Azis, Ioanna Zerva, Paraschos Melidis, Carlos Caceres, Kostas Bourtzis, Spyridon Ntougias

**Affiliations:** 1 Laboratory of Wastewater Management and Treatment Technologies, Department of Environmental Engineering, Democritus University of Thrace, Vas. Sofias 12, 67100 Xanthi, Greece; 2 Insect Pest Control Laboratory, Joint FAO/IAEA Programme of Nuclear Techniques in Food and Agriculture, A-1400, Vienna, Austria

**Keywords:** Biomass valorization, Insect mass rearing, Agricultural wastes

## Abstract

**Background:**

*Enterobacter* sp. AA26 was recently isolated from the midgut of *Ceratitis capitata* (Wiedemann) and it was shown to have positive effects in rearing efficiency when used as larval probiotics. In this study, biomass production was carried out in bench-scale bioreactors to elucidate the biokinetic properties of *Enterobacter* sp. AA26 and its nutritional value.

**Results:**

Strain AA26 is a psychrotolerant, halotolerant, facultatively anaerobic bacterium with broad pH range for growth (pH 4 to 10.2), which possessed the typical biochemical profile of *Enterobacter* spp. The specific oxygen uptake rate (SOUR) was calculated as 63.2 ± 1.26 and 121 ± 1.73 mg O_2_ g^− 1^ VSS h^− 1^, with the yield coefficients in acetate and glucose being equal to 0.62 ± 0.03 and 0.67 ± 0.003 g biomass produced/g substrate consumed, respectively. The maximum specific growth rate (μ_max_) of strain AA26 grown in fill-and-draw bioreactors at 20 °C and 35 °C was 0.035 and 0.069 h^− 1^, respectively. Strain AA26 grew effectively in agro-industrial wastewaters, i.e. cheese whey wastewater (CWW), as alternative substrate for replacing yeast-based media. Biomass of strain AA26 could provide all the essential amino acids and vitamins for the artificial rearing of *C. capitata*. Greater intracellular α- and β-glucosidase activities were observed during growth of strain AA26 in CWW than in yeast-based substrate, although the opposite pattern was observed for the respective extracellular activities (*p < 0.01*). Low protease activity was exhibited in cells grown in yeast-based medium, while no lipase activities were detected.

**Conclusions:**

The ability of strain AA26 to grow in agro-industrial wastes and to provide all the essential nutrients can minimize the cost of commercial media used for mass rearing and large scale sterile insect technique applications.

## Background

The Mediterranean fruit fly *Ceratitis capitata* (Wiedemann), commonly named as medfly, is one of the major insect pests worldwide. This polyphagous pest negatively affects fruit production since oviposition of adult female medflies and larvae development under the fruit skin can result in serious crop damage [1]. The sterile insect technique (SIT) has been broadly adopted to combat this destructive pest. SIT is based on the mass production and release of irradiated sterile insects. Through continuous releases of overflooding ratios of sterile insects, wild females mate with the sterile males and the target population is suppressed [2].

During the recent years, it has been shown that insects have established sophisticated symbiotic associations (e.g. parasitic or mutualistic relationships) with diverse microorganisms, including bacterial species [3, 4]. These symbiotic bacteria play a catalytic role in the biology, physiology, ecology and evolution of insect species, affecting nutrition, immunity, mating behavior, reproduction and pest status of their hosts [3, 4].

The structure and properties of the gut-associated microbiota of medfly *Ceratitis capitata* has recently been studied [5–7]. The medfly gut was found to be dominated almost exclusively by representatives of the family *Enterobacteriaceae*. In particular, members of *Klebsiella*-*Enterobacter*-*Citrobacter* group, the ex *Enterobacter* linkage *Pantoea* genus, and *Pectobacterium* spp. were the predominant taxa in the gut of *Ceratitis capitata* [5–7]. The predominance of such pectinolytic and diazotrophic population appears to influence medfly’s diet and fitness [5, 6, 8]. Additional studies in medfly have also indicated that irradiation-induced dysbiosis can be potentially restored by enhancing male sexual performance through *Klebsiella oxytoc*a probiotic applications [7]. *Enterobacter agglomerans*, *Klebsiella pneumoniae* and other bacterial isolates have also been used in adult probiotic applications under laboratory conditions [9, 10]. The ability of *Enterobacter*-*Klebsiella* group members to colonize the gut biofilm of the sterile males makes advantageous their application as probiotic bacteria in mass rearing and SIT applications [11].

Recently, the gut-associated symbiont *Enterobacter* sp. AA26 was isolated from the *Ceratitis capitata* Vienna 8^D53+^ genetic sexing strain (GSS) and was shown to improve the productivity of this strain [12]. To assess the potential of this symbiont in the artificial diet of medfly under mass rearing conditions and its potential cost-effectiveness for large scale operational SIT programmes, large quantities of biomass are required. The present study investigates the biokinetic properties of *Enterobacter* sp. AA26 for mass production of biomass in full-scale bioreactors towards its potential use in mass rearing facilities and large scale applications. Indeed, mass production of *Enterobacter* sp. AA26 from low-cost agricultural residues, which are easily biodegradable and accessible worldwide, such as cheese whey wastewater, can substitute Torula yeast (syn. *Candida utilis*), minimizing thus the cost of purchasing this ingredient that is widely used for mass rearing in SIT applications.

## Methods

*Enterobacter* sp. AA26 was isolated from the gut of the medfly Vienna 8^D53+^ GSS as described previously [12]. The identity of the biological material used in all tests described below was confirmed by sequencing the 16S rRNA gene, which was found identical to that previously reported [12].

### Physiological and biochemical characteristics of *Enterobacter* sp. AA26

The biochemical profile of *Enterobacter* sp. AA26 was examined by using the EnteroPluri kit (BD, USA), following manufacturer’s instructions. Pectinase activity was examined by using pectinase screening agar medium, consisting of 1% w/v citrus pectin, 0.14% w/v (NH_4_)_2_SO_4_, 0.6% w/v K_2_HPO_4_, 0.2% w/v KH_2_PO_4_ and 0.01% w/v MgSO_4_.7H_2_O in the presence of 1.7% w/v agar [13]. Pectin-containing agar plates in the presence of 0.10% w/v yeast extract were also prepared. Catalase and oxidase reactions were performed according to Smibert and Krieg [14].

The pH range for growth was investigated by using a nutritional base consisting of 10 g L^− 1^ peptone and 5 g L^− 1^ yeast extract, supplemented with the appropriate buffer solution. The following pH values were tested: pH 3 (adjusted by citric acid addition), pH 4 (0.06 M citric acid - 0.04 M citrate), pH 5 (0.035 M citric acid - 0.065 M citrate), pH 6 (0.013 M Na_2_HPO_4_–0.087 M KH_2_PO_4_), pH 7 (0.061 M Na_2_HPO_4_–0.039 M KH_2_PO_4_), pH 8 (0.095 M Na_2_HPO_4_–0.005 M KH_2_PO_4_), pH 9 (0.1 M NaHCO_3_–1 mM K_2_HPO_4_), pH 10.2 (0.075 M Na_2_CO_3_–0.025 M NaHCO_3_–1 mM K_2_HPO_4_) and pH 11 (0.1 M Na_2_CO_3_) [15]. The salt range for growth was investigated by using the above nutritional base in the presence of 0, 1, 3, 5, 8, 9, 10 and 11% w/v NaCl. LB (Luria-Bertani) media were used to investigate the temperature range for growth of strain AA26 (4, 7, 11, 15, 20, 25, 30, 37, 40 and 43 °C were tested). Anaerobic growth was examined by using the Anaerocult A anaerobic system (Merck, Germany). All the above media were solidified with 17 g L^− 1^ agar.

### Determination of growth characteristics of *Enterobacter* sp. AA26 in batch cultures

Growth curves were constructed by measuring the optical density at 600 nm (OD_600 nm_) after inoculation of LB liquid media with strain AA26 at 30 °C. In addition, growth of *Enterobacter* sp. AA26 was examined in 1:5 v/v cheese whey wastewater (10,000 mg/L final Chemical Oxygen Demand - COD concentration, with pH being adjusted at 7). *Enterobacter* sp. AA26 was also cultivated in the following growth media: I) 10 g L^− 1^ peptone, II) 10 g L^− 1^ peptone and 10 g L^− 1^ NaCl, III) 10 g L^− 1^ glucose and 0.2 g L^− 1^ yeast extract and IV) 10 g L^− 1^ glucose.

### Biokinetic parameters of *Enterobacter* sp. AA26 in fill and draw bioreactors

Samples were obtained aseptically to determine protein biomass content during bioreactor operation. In details, the obtained biomass was centrifuged at 10,000 g for 5 min (at 4 °C), washed in 20 mM Tris-HCl (pH 7.6) and disrupted on ice for 15 min (at a pulse of 0.6 s with 0.4-s interval, 50% duty cycle) by using a Hielscher UP200S sonicator. The homogeneous biomass was centrifuged (15,000 g at 4 °C for 15 min) and the cell-free lysate was collected for protein determination. Protein content was quantified by the Bradford method [16].

Laboratory-scale bioreactors of 1 L each (working volume of 0.6 L) were fed with LB broth and inoculated aseptically with the medfly gut symbiont *Enterobacter* sp. AA26. The strain AA26 was grown under the fill and draw mode and the biomass growth characteristics were determined.

**Fig. 1:**
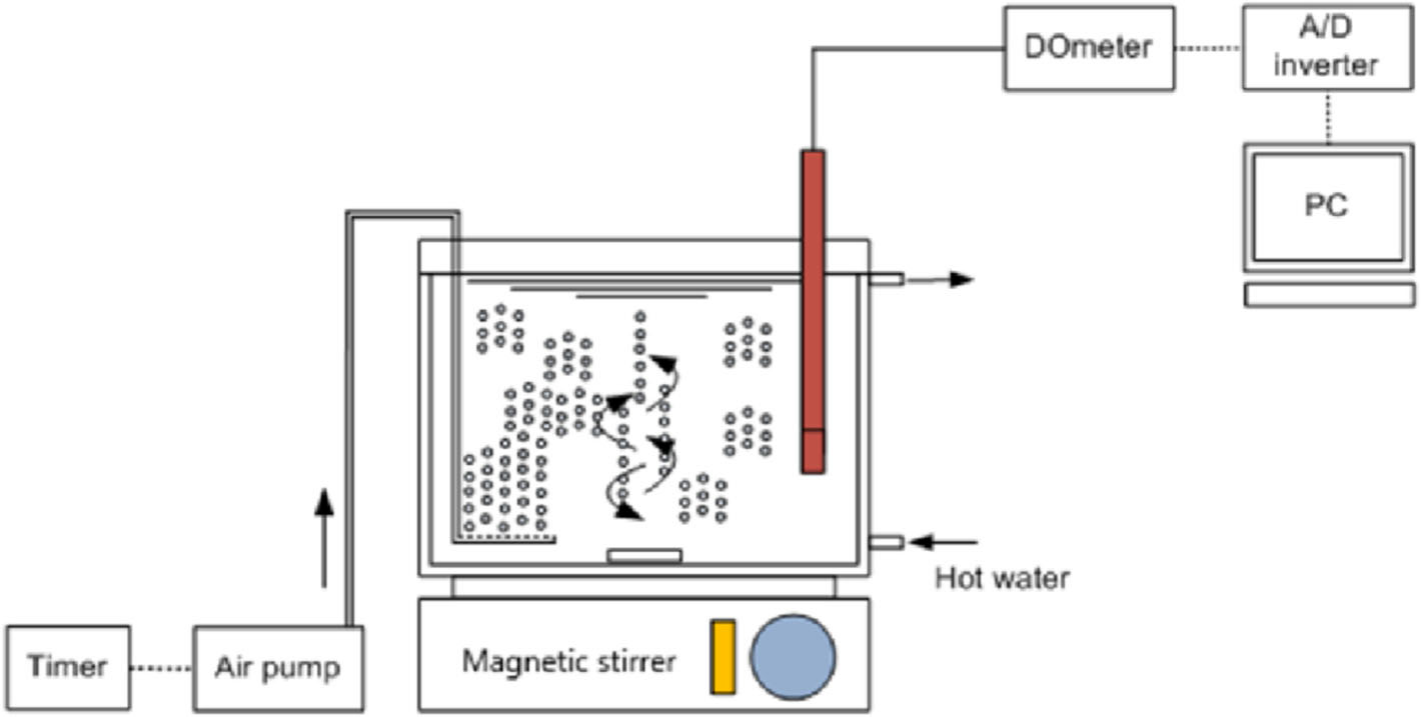
Schematic layout of the unit used for oxygen uptake rate (OUR) measurements

Oxygen uptake rate (OUR) was determined in 1 L working volume sterile bioreactor (1.2 L in total), where dissolved oxygen was online measured using a WTW (Wissenschaftlich-Technische Werkstätten) dissolved oxygen (DO) meter. The DO meter was connected to a computer and measurements were obtained every 15 s. An air pump was used to achieve adequate aeration (approximately 7 mg/L) and cells agitation was performed (Fig. 1). Aeration of the culture was interrupted and the resulting decrease in the oxygen concentration was recorded as a function of time. Aeration/non-aeration cycles of 12 min were performed, consisting each one of 6 min aeration-on and 6 min aeration-off periods. OUR (mg O_2_ L^− 1^ h^− 1^) was estimated by determining the slope during linear decline of DO and SOUR (expressed as mg O_2_ g^− 1^ VSS h^− 1^) was measured by dividing OUR by the volatile suspended solids (VSS) present in the bioreactor. After passing the culture into endogenous respiration, acetate or glucose was added and both OUR and SOUR were calculated. The VSS concentration was determined as described in Standard Methods for the Examination of Water and Wastewater [17] to determine the specific oxygen uptake rate (SOUR). The yield coefficient (Y_H_), i.e. g biomass produced/g substrate consumed, was calculated according to the following formula [18, 19]:

$$ {\mathrm{Y}}_{\mathrm{H}}=1-\frac{OU}{S_S} $$

where ΟU (mg O_2_ L^− 1^) is the oxygen consumed for the complete oxidation of organic substrate added (estimated by calculating the cumulative oxygen consumption area), and S_S_, the COD concentration of the biodegradable substrate added (mg O_2_ L^− 1^).

The specific growth rate (μ) of strain AA26 was determined by calculating the dilution rate (D) at steady operational conditions. The dilution rate can be defined by dividing the flow rate of the medium entered by the volume of the culture in the bioreactor [20]:

$$ \upmu =\mathrm{D}=\frac{\mathrm{medium}\kern0.17em \mathrm{flow}\kern0.17em \mathrm{rate}}{\mathrm{culture}\ \mathrm{volume}} $$

The maximum specific growth rate (μ_max_) was determined by estimating the maximum broth flow rate, without the strain being washed-out.

### Determination of amino acid and vitamin compositions

Amino acid and vitamin analyses were performed in Analytical Laboratories of Athens S. A. (Greece). Extraction of water soluble vitamins was carried out in 10 mM ammonium acetate solution, pH 4.5, through ultrasonic homogenization and deproteinization with 10% w/v trichloroacetic acid, while fat soluble vitamins were obtained after saponofication with ethanolic base (2% w/v NaOH) and successive hexane extractions. Both water and fat soluble vitamins were analyzed in a TSQ Quantum Access MAX Triple Quadrupole Mass Spectrometer equipped with a 50 × 2 mm Phenomenex Synergi Fusion-RP LC column (2.5 μm and 100 Å particles and pores size, respectively). Amino acids extraction was performed by adding 6 M HCl and 0.5% w/v phenol and placing the analyzed samples in a microwave oven. Detection of amino acids was carried out in a HILIC-ESI-MS-MS system (Thermo Scientific). Protein determination in Torula yeast was performed by estimating the total Kjeldahl nitrogen (TKN) of the samples and multiplying their TKN content by a conversion factor of 6.25 (AOAC 2001.11 method).

### Quantification of hydrolytic activities of *Enterobacter* sp. AA26

Protease, lipase, α- and β-glucosidase activities were determined by growing strain AA26 in both LB and 1:5 v/v cheese whey wastewater and obtaining their cell-free lysate and supernatant (broth), respectively. Extracellular and intracellular proteolytic and lipolytic activities were determined based on the protocols reported in Gessesse et al. [21], while the protocol used for the estimation of the respective α- and β-glucosidase activities was based on Benitez et al. [22] protocol as modified by Ntougias [23]. The Student’s t-test was used to comparatively examine the treatment means of the enzyme activities.

Proteolytic activity was measured using 0.5% w/v azocasein in 20 mM Tris-HCl. A quantity of 800 μL azocasein was mixed with 200 μL of lysate and incubated at 30 °C for appropriate time period (up to 1 day dependent on the sample examined). After addition of 500 μL 15% w/v trichloroacetic acid and 30 min-incubation, the mixture was centrifuged at 14,000 g and 800 μL of the clear supernatant were mixed with 200 μL 2 N NaOH. Protease activity was determined by monitoring the absorbance at 440 nm against a blank.

Estimation of α- and β-glucosidase activities were determined by using 0.05 M 4-nitrophenyl-α-D-glucanopyranoside or 4-nitrophenyl-b-D-glucanopyranoside, respectively. A quantity of 0.4 mL 4-nitrophenyl-D-glucanopyranoside (α- or β-, as appropriate) was mixed with 1 mL lysate in the presence of 0.6 mL 0.02 M Tris-HCl and incubated for appropriate time period. Glucosidase activity was determined by measuring the absorbance at 410 nm against a blank.

Lipase activity was determined by using 20 mM p-nitrophenol palmitate as the stock solution. A working solution was made by adding 2.5 mL stock solution, 0.05 g gum Arabic, 0.2 mL Triton and 47.5 mL 20 mM Tris-HCl solution (pH 8). A quantity of 2.7 mL working solution was mixed with 0.3 mL lysate and incubated for appropriate time period. Lipase activity was determined by measuring the absorbance at 410 nm against a blank.

## Results

### Physiological and biochemical traits of *Enterobacter* sp. AA26

*Enterobacter* sp. AA26 could grow under a broad pH range, i.e. from pH 4 to pH 10.2. No growth of strain AA26 was observed at pH 3 or pH 11. Appearance of colonies was observed at day 1 after inoculation at any pH grown, indicating that strain AA26 is a neutrophile with a broad pH range for growth (both acidic and alkaline).

Isolate AA26 could grow both in the absence of salt and in the presence of NaCl concentration up to 10% w/v, showing optimum growth at salinities within 0–8% w/v NaCl. No growth was observed at salt concentration of 11% w/v NaCl. Therefore, strain AA26 is a halotolerant bacterium, growing up to 10% w/v NaCl.

Strain AA26 could grow at a temperature range of 4 to 40 °C with a broad optimum for growth of 25–40 °C, while no growth was observed at 43 °C. Based on these findings, strain AA26 can be characterized as psychrotolerant bacterium. Bacterial isolate AA26 could grow in the presence and absence of oxygen; therefore, it is a facultatively anaerobic bacterium.

In addition, growth under aerobic conditions was also detected in the presence of 10 g L^− 1^ peptone only (without yeast extract and NaCl addition) as well as in medium consisting of 10 g L^− 1^ peptone and 10 g L^− 1^ NaCl. No aerobic growth was observed in medium consisting of 10 g L^− 1^ glucose only, while growth was restricted in the presence of 10 g L^− 1^ glucose and a limited amount (0.2 g L^− 1^) of yeast extract.

Based on EnteroPluri profile, strain AA26 exhibited the biochemical pattern presented in Table 1. *Enterobacter* sp. AA26 could ferment adonitol, glucose, lactose and sorbitol, hydrolyze urea, decarboxylate ornithine, utilize citrate and produce acetoin, but gave negative reactions for lysine decarboxylation, hydrogen sulphide production, tryptophan bioconversion to indole, phenylalanine deamination, and arabinose and dulcitol fermentation. Moreover, strain AA26 was oxidase-negative and catalase-positive, giving a strong catalase reaction. No growth was observed in the pectin-based medium in the absence and presence of yeast extract, indicating that no pectinase activity was induced by *Enterobacter* sp. AA26.

**Table 1:** Biochemical profile of *Enterobacter* sp. AA26 using EnteroPluri diagnostic kit

Biochemical reactions	
Glucose fermentation/gas production in anaerobiosis	positive/positive
Lysine decarboxylation in anaerobiosis	negative (slightly green)
Ornithine decarboxylation in anaerobiosis	positive
Hydrogen sulphide production/indole test	negative/negative
Adonitol fermentation	positive
Lactose fermentation	positive
Arabinose fermentation	negative
Sorbitol fermentation	positive
Acetoin production (Voges-Proskauer)	positive
Dulcitol fermentation/phenylalanine deamination	negative/negative
Urea hydrolysis	positive
Citrate utilization	positive

### Biokinetic properties of *Enterobacter* sp. AA26

**Fig. 2:**
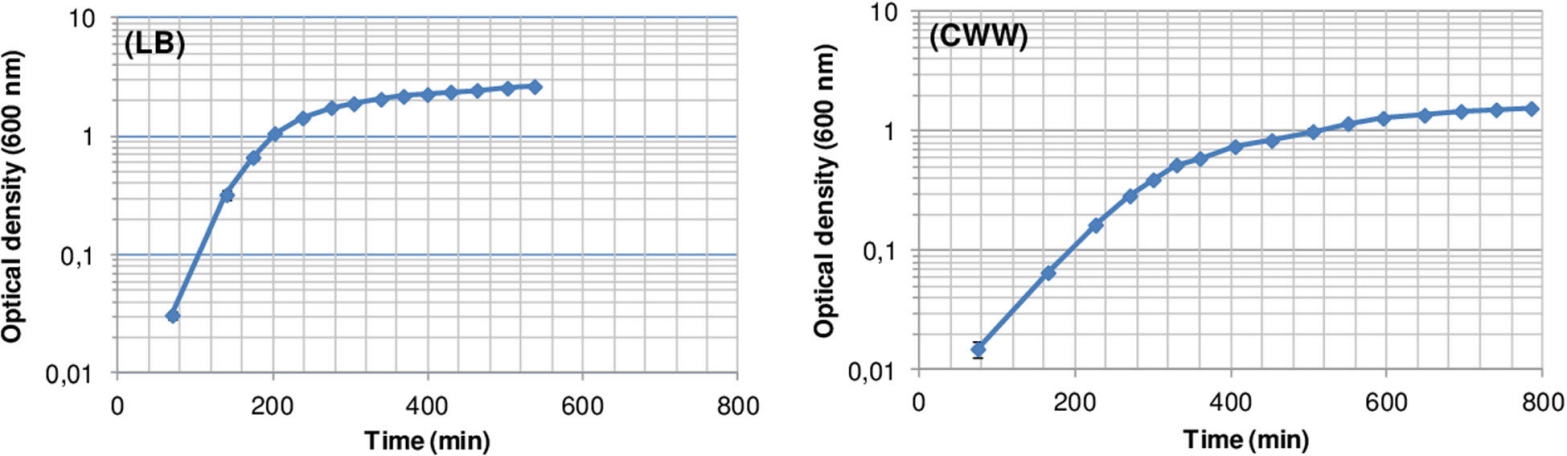
Growth curves of *Enterobacter* sp. AA26 during cultivation in LB broth (left) and CWW (right) (*n* = 3)

The growth curves of strain AA26 cultivated in LB and CWW under the batch mode are shown in Fig. 2. Based on Fig. 2, the double time (t_d_) of the strain was 20 min and 42 min in LB and CWW, which corresponded to specific growth rates (μ) of 2.08 h^− 1^ and 0.99 h^− 1^, respectively. The biomass produced was estimated at the late exponential phase as 2145 ± 56 mg dry weight/L LB (*n* = 3), whereas its protein content was determined as 56.6 ± 6.3% (n = 3).

By cultivating *Enterobacter* sp. AA26 in a fill and draw bioreactor under sterile conditions, the maximum specific growth rate (μ_max_) of strain AA26 growing in LB at 20 °C and 35 °C was determined by calculating the maximum dilution rate (D_max_), in which the bioreactor was operated under stable conditions without the biomass being wash-out. These growth rates were estimated to be 0.035 h^− 1^ and 0.069 h^− 1^, respectively. The protein content of the dry biomass under the fill and draw operating conditions was calculated as 53.7 ± 1.2% (*n* = 3). The respective content in Torula yeast was determined as 44.2 ± 0.4%.

**Fig. 3:**
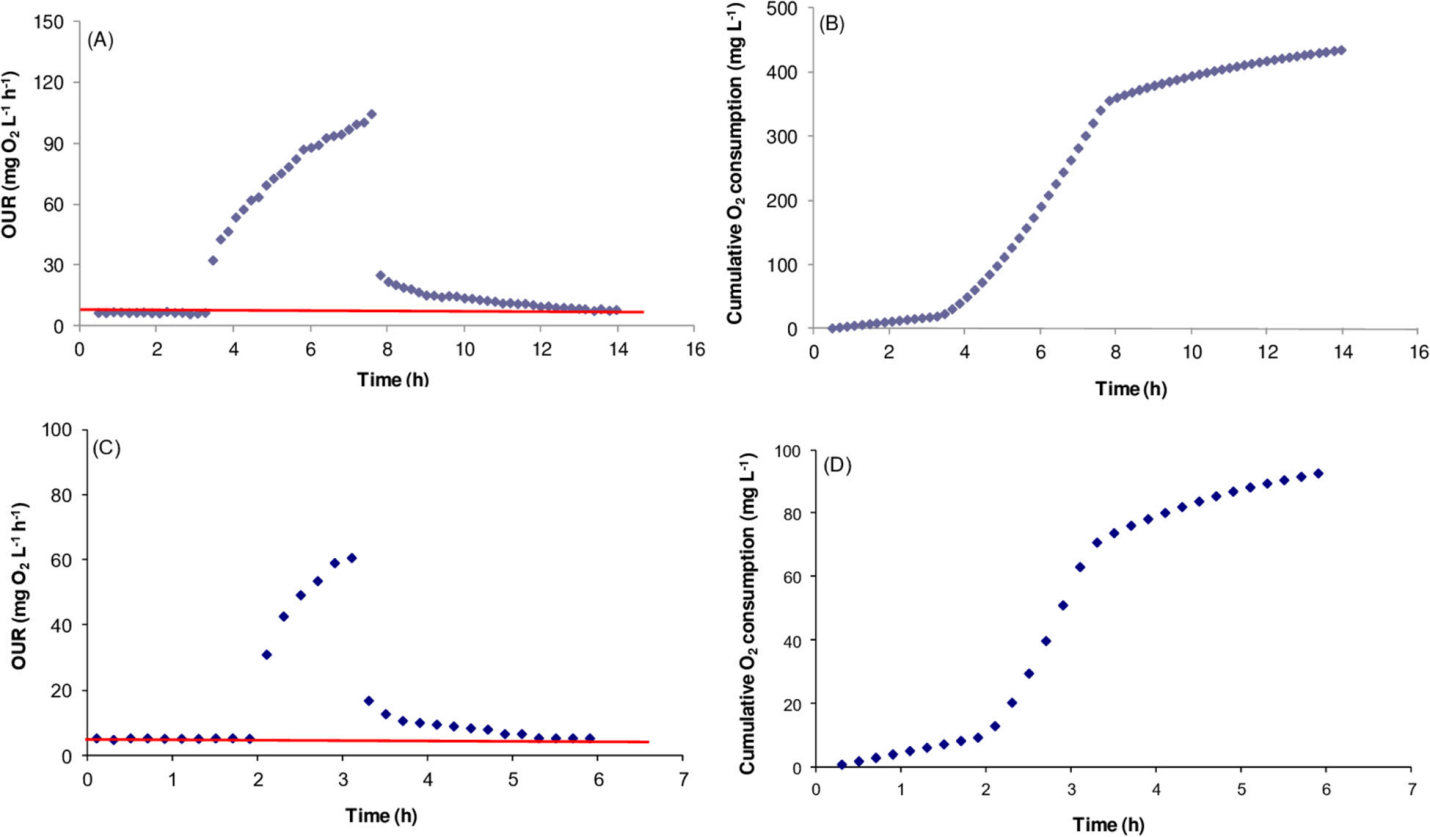
Profiles of oxygen uptake rate (**a** and **c**, for acetate and glucose, respectively) and cumulative O_2_ consumption (**b** and **d**, for acetate and glucose respectively) in *Enterobacter* sp. AA26

Oxygen uptake and specific oxygen uptake rates were calculated by using acetate and glucose as the substrate consumed (Table 2 and Fig. 3). The oxygen uptake rates and the specific oxygen uptake rates were determined as equal to 79.6 ± 1.59 mg O_2_ L^− 1^ h^− 1^ and 63.16 ± 1.26 mg O_2_ g^− 1^ VSS h^− 1^ as well as 71.4 ± 6.76 mg O_2_ L^− 1^ h^− 1^ and 121 ± 1.73 O_2_ g^− 1^ VSS h^− 1^ for acetate and glucose, respectively. Based on the formula ${\mathrm{Y}}_{\mathrm{H}}=1-\frac{OU}{S_S}$, the yield coefficients were calculated to be 0.62 ± 0.03 and 0.67 ± 0.003 g biomass produced/g substrate consumed by using acetate and glucose as the substrates consumed (Table 3).

**Table 2:** Oxygen uptake rate (OUR) and specific oxygen uptake rate (SOUR) of *Enterobacter* sp. AA26 cells. The food to microorganism ratio was set at 0.2 g substrate g^−1^ VSS d^− 1^ immediately after interrupting the aeration

	Replicates	OUR (mg O_2_ L^− 1^ h^− 1^)	SOUR (mg O_2_ g^− 1^ VSS h^− 1^)
Acetate	1	78.5	62.3
	2	82.7	65.6
	3	77.5	61.5
	Mean ± SE	79.6 ± 1.59	63.2 ± 1.26
Glucose	1	69.6	118
	2	83.9	142
	3	60.7	103
	Mean ± SE	71.4 ± 6.76	121 ± 1.73

**Table 3:** Determination of yield coefficient (Y_H_) of *Enterobacter* sp. AA26

	Replicates	Y_H_ (g biomass produced/g substrate consumed)
Acetate	1	0.59
	2	0.68
	3	0.60
	Mean ± SE	0.62 ± 0.03
Glucose	1	0.67
	2	0.66
	3	0.67
	Mean ± SE	0.67 ± 0.003

### Comparison of amino acid and vitamin compositions of *Enterobacter* sp. AA26 and Torula yeast

**Fig. 4:**
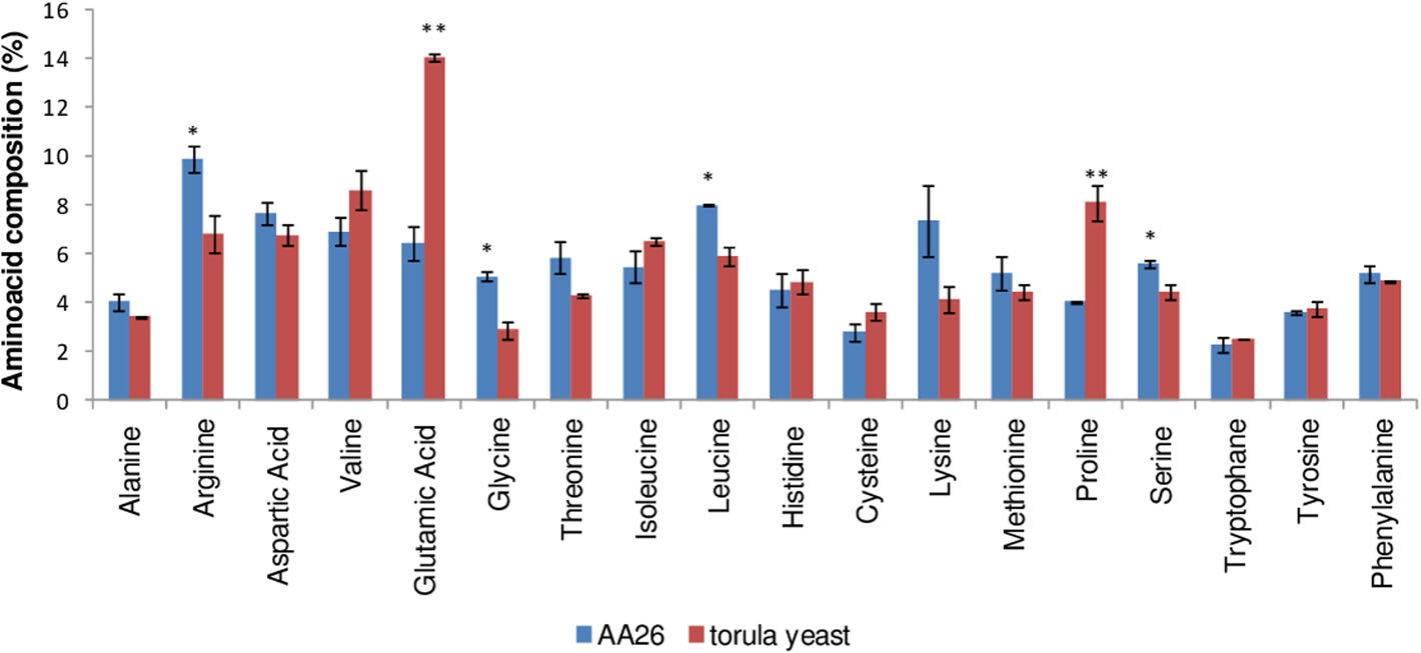
Amino acid composition of *Enterobacter* sp. AA26 and Torula yeast

*Enterobacter* sp. AA26 could synthesize all the essential amino acids, possessing an amino acid composition consisted mainly of arginine, aspartic acid, leucine and lysine, which represented 33% of its protein content (Fig. 4). Torula yeast, which is one of the main ingredients in the artificial diet of medfly, also contained all the essential amino acids, although in this case, glutamic acid, valine and proline were the major amino acids detected, covering 31% of the yeast’s protein content (Fig. 4). Statistically greater glutamic acid and proline content (*p < 0.01* in Student t-test) was detected in *Candida utilis* compared to *Enterobacter* sp. AA26 cells, whereas the percentages of arginine, glycine, leucine and serine were greater (*p < 0.05* in Student t-test) in *Enterobacter* sp. AA26 cells than in Torula yeast (Fig. 4).

**Fig. 5:**
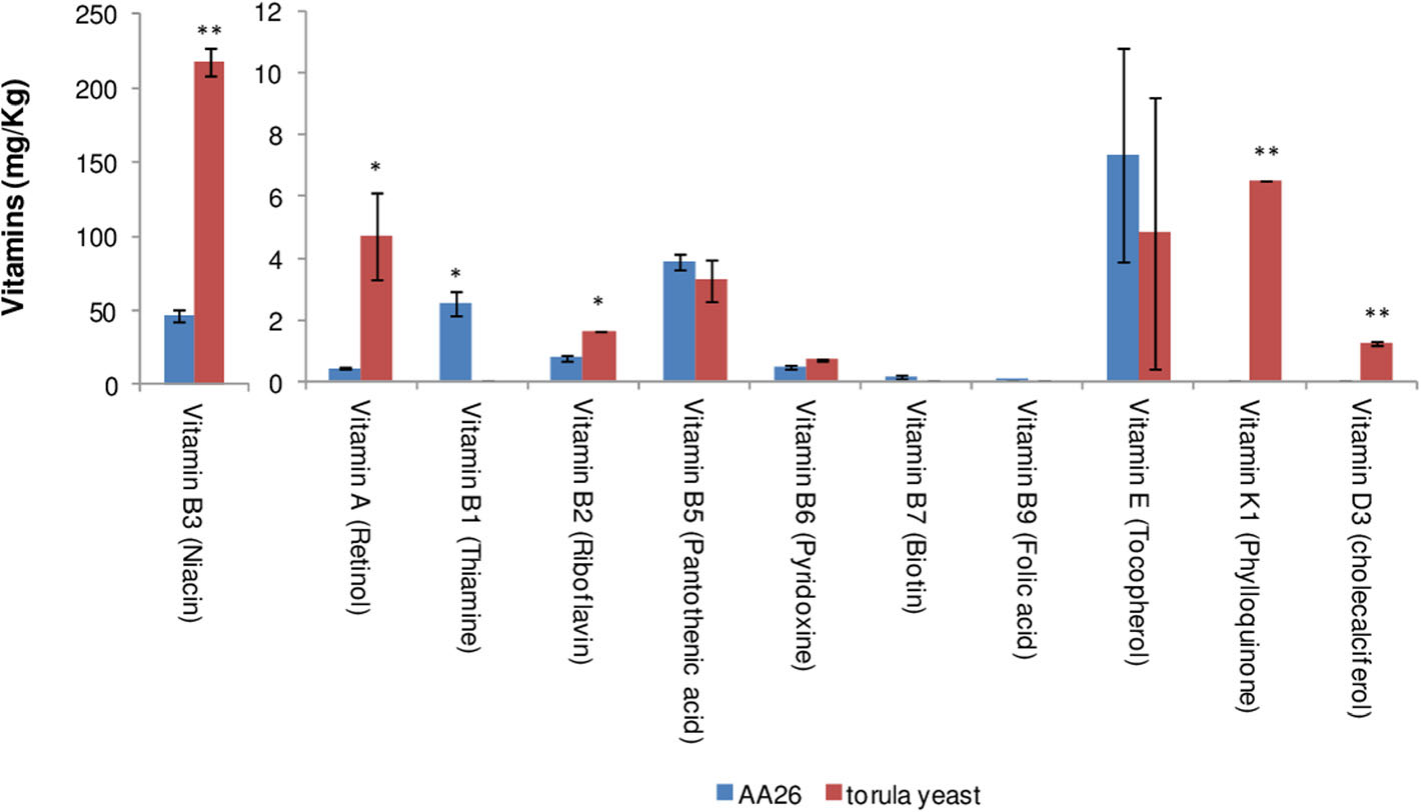
Vitamin composition of *Enterobacter* sp. AA26 and Torula yeast. Vitamins B12, C, D3, K1 and K3 in strain AA26 and vitamins B1, B7, B9, B12, C and K3 in Torula yeast were below the detection limit. The detection limits of vitamins B1, B7, B9, B12, C, D3, K1 and K3 were 0.013, 0.01, 0.023, 0.1, 7, 0.01, 0.02 and 0.01 mg/Kg, respectively. Vitamin contents were compared by Student’s t-test, apart from the vitamins B7 and B9, which were marginally above the detection limit in the case of AA26 cells

Niacin was the major vitamin detected in *Enterobacter* sp. AA26 and *Candida utilis* cells. However, niacin in Torula yeast was approximately 5 fold greater than the respective content in strain AA26 (Fig. 5). Νo substantial differences between *Enterobacter* sp. AA26 and Torula yeast were found for vitamin B5, B6, B7, B9 and E, while greater vitamin A, B2, K1 and D3 content was determined for Torula yeast as compared to *Enterobacter* sp. AA26 (Fig. 5). Vitamins B1, B7 and B9 were only detected in AA26 cells, but only thiamine was found in reasonable amount (Fig. 5).

### Hydrolytic activities of *Enterobacter* sp. AA26 grown in yeast-based media and cheese whey wastewaters

**Fig. 6:**
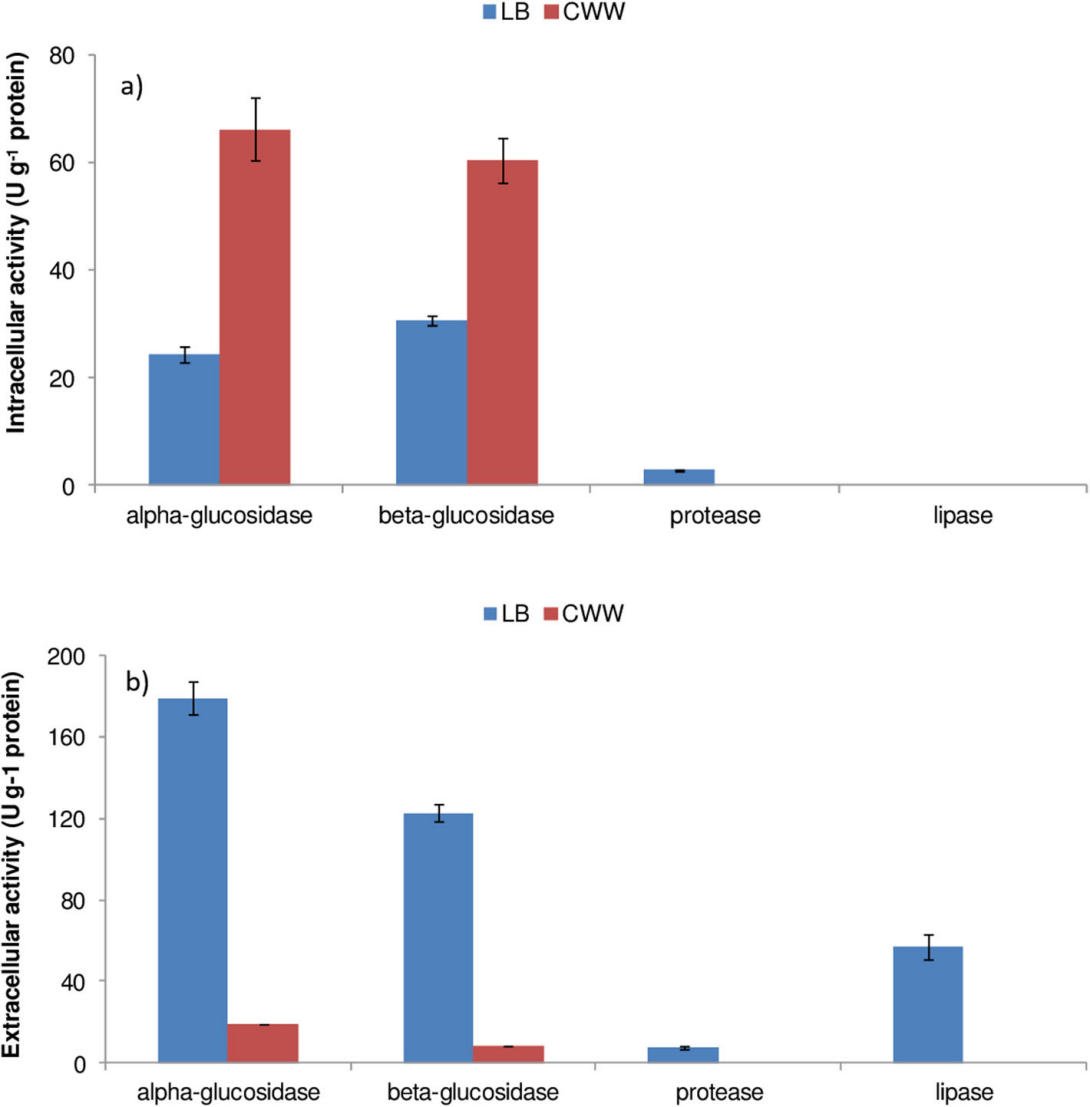
Intracellular (**a**) and extracellular (**b**) hydrolytic activities of *Enterobacter* sp. AA26 cultivated in commercial available yeast-based medium and cheese whey wastewater

The glucosidase, protease and lipase activities of the lysates of strain AA26 cultivated in LB and CWW are presented in Fig. 6. Greater α- and β-glucosidase activities were observed during growth of strain AA26 in CWW, while the respective activities were almost half in the case of growth in LB broth (*p < 0.01* in Student’s t-test). Moreover, low protease activity was exhibited by the “LB” lysate, while no lipase activities were detected in the lysates derived from the growth of strain AA26 in LB and CWW. No statistical significant differences were found between α- and β- glucosidase activities determined in “CWW” lysate.

In comparison to CWW where low extracellular glucosidase activities were detected, significant higher extracellular enzyme activities were observed when *Enterobacter* sp. AA26 grew in LB medium (*p < 0.01* in Student’s t-test) (Fig. 6). However, α-glucosidase activity was greater than the β-glucosidase activity determined during growth of strain AA26 in both LB broth and CWW (*p < 0.01* in Student’s t-test) (Fig. 6).

## Discussion

The limited number of technologies to adequately produce novel biocontrol and probiotic agents is the main obstacle to their biotechnological application. A range of parameters, such as cultivation method and conditions, storage, formulation and reconstitution process, should be extensively examined for real-scale applications [24]. A few studies have been performed on the cultivation of *Enterobacter* spp., which is mainly restricted to hydrogen production from the fermentation of wastes [25, 26] and exopolysaccharide production [27]. No biocontrol or probiotic agent belonging to the genus *Enterobacter* has been cultivated in bioreactors for biotechnological purposes and the investigation of biomass characteristics is a challenging task.

Based on the examination of physiological characteristics, *Enterobacter* sp. AA26 is a psychrotolerant, halotolerant, facultatively anaerobe with broad growth pH range. The ability of this isolate to grow under a wide pH range and high salt concentrations indicates that strain AA26 can be effectively adapted at various habitats. Thus, substrates of high salinity and/or low or high pH like several agro-industrial wastewater can be considered as potential low-cost alternative growth media. The inability of the strain to grow over 40 °C may negatively affect its use as probiotic agent at high ambient temperatures, which is, however, not the case for its insect host *Ceratitis capitata*.

On the other hand, *Enterobacter* sp. AA26 could utilize peptone as the sole carbon source for growth, a fact that reduces the cost of cultivation since no yeast extract addition is required. This is in accordance with the findings of Potrikus and Breznak [28] who reported that peptone was an ideal nitrogen source for the cultivation of *Enterobacter agglomerans* strains C-1 and C-2. Moreover, pectinolytic population has been reported to influence medfly’s diet and fitness [8]. However, no pectinase activity was exhibited by *Enterobacter* sp. AA26, indicating that this medfly gut-associated symbiont did not possess a mode of action that is linked to pectin degradation.

As a member of the genus *Enterobacter*, strain AA26 fermented lactose and glucose, producing gas, and it was oxidase-negative, indole-negative and Voges-Proskauer-positive. Moreover, it decarboxylated ornithine, lacked phenylalanine deaminase activity and did not produce H_2_S. As a typical facultative anaerobe, *Enterobacter* sp. AA26 was oxidase-negative, which exerted a strong catalase reaction to tolerate the oxidative stress induced by oxygen species.

*Enterobacter* sp. AA26 exhibited among the highest specific growth rates ever reported for *Enterobacter cloacae* strains cultivated in yeast-based [29] and sugar-based [30] media under the batch mode. On the other hand, greater dilution rates for *E. cloacae* WD7 were detected [31]. Moreover, the yield coefficient (Yx/s) of strain WD7 was estimated as 0.03 g cells/g sucrose, which is much less than that calculated for strain AA26.

*Enterobacter* sp. AA26 and Torula yeast differed greatly in the proportion of glutamic acid and proline, although smaller differences were identified in the percentages of arginine, glycine, leucine and serine (Fig. 4). It is well known that amino acid effects on insect fitness are dose- and type-dependent [32, 33]. In comparison to known amino acid-rich medfly larval and adult diets [34], glutamic acid and proline represented 6.5 and 4.1% of the protein content in strain AA26, while the respective percentages in defined diets were 21.5 and 7.8%, respectively [34]. All the other amino acids were in greater proportion in strain AA26 as compared to the defined diets. In general, both glutamic acid and proline are considered as non-essential amino acids for insect species [33]. *C. capitata* larvae reared without glutamic acid and/or proline could be developed in a similar manner with that fed with a complete medfly meridic diet, although adverse effects have been reported from the absence of such amino acids in *C. capitata* adult diet [34]. Moreover, lack of threonine and tryptophan in medfly diet has been reported to induce severe effects on insect development, with no larvae survival within a period of 18 days [34]. However, these essential to medfly amino acids were in adequate proportion in strain AA26 and similar to those found in Torula yeast. The amino acids glycine and serine, which play key role in medfly fitness, were also in greater proportions in strain AA26 than in Torula yeast [35].

Niacin was the major vitamin detected in both *Enterobacter* sp. AA26 and Torula yeast [36]. Despite the fact that niacin in Torula yeast analyzed was approximately 5-fold greater than the respective content in strain AA26, other yeasts and yeast-based products used for mass rearing contained similar to strain AA26 niacin content [36]. This indicates that the use of *Enterobacter* sp. AA26 biomass in medfly diet can provide all the required vitamins.

Distinct enzyme profiles were obtained for strain AA26 when it grew in LB broth and CWW, a fact that may influence the effects of strain AA26 on medfly diet and attractiveness. In particular, greater intracellular rather than exocellular α- and β-glucosidase activities were determined during growth of strain AA26 in CWW, while the opposite trend was observed in LB broth. The high exopolysaccharides content secreted by *Enterobacter cloacae* strains [31] may be responsible for the high extracellular glucosidase activities detected in LB medium during growth of strain AA26. In addition, the greater intracellular as compared to exocellular glucosidase activities during growth in CWW indicate that CWW carbohydrates (mainly glucose and lactose) were easily accessible in the cytosol. Lactose carriers have been found in phylogenetic relatives of enterobacteria, i.e. *Klebsiella* and *Citrobacter* strains [37, 38], and in the genome of *Enterobacter cloacae* UW5 (GenBank Accession number NZ_CP011798). On the other hand, strain AA26 was capable of growing effectively in agricultural wastewaters (t_d_ of 42 min in CWW) and therefore, use of such organic substrate can replace the costly substrates used for the preparation of LB, i.e. commercial yeast and peptone. Regarding CWW, *Enterobacter* spp. have been used in producing biohydrogen during dark fermentation of this agro-industrial effluent [39], whereas the bioconversion of cheese whey by *Enterobacter* sp. A47 to the bioactive compounds glucuronic acid and fucose, which can be used in potential high-value nutraceutical and pharmaceutical applications, was recently reported [40]. Moreover, as shown in Fig. 6, *Enterobacter* sp. AA26 isolated from the midgut of *Ceratitis capitata* exerted high *β*-glucosidase (cellobiase) activities. The induction of cellobiases has been reported to be favored in the midgut of insect species [41]. Interestingly, Anand et al. [42] isolated an *Enterobacter* sp. from the gut of *Bombyx mori* that exhibited high *β*-glucosidase activity, reporting that most *B. mori* disaccharidases have been found in the midgut tissues. Possible role of such microbe on cellulose degradation of the fruit biomass digested by *Ceratitis capitata* cannot be excluded.

## Conclusions

*Enterobacter* sp. AA26 was capable of being cultivated under broad environmental conditions and could grow effectively in both commercial yeast-based media and agricultural wastewaters by implementing the batch and the fill-draw mode of operation. The replacement of peptone and yeast, which commonly used in commercial media, with alternative organic substrates like agro-industrial wastes may potentially minimize the cultivation cost in full-scale insect mass rearing facilities. Moreover, *Enterobacter* sp. AA26 as a probiotic strain is capable of providing the entire spectrum of both essential and non-essential amino acids and vitamins in adequate amount for medfly mass rearing and sterile insect technique applications.

## Data Availability

All data is included in the manuscript.

## Abbreviations

CWW: Cheese whey wastewater
DO: Dissolved oxygen
GSS: Genetic sexing strain
OUR: Oxygen uptake rate
SIT: Sterile insect technique
SOUR: Specific oxygen uptake rate
t_d_: Double time
VSS: Volatile suspended solids
Y_H_: Yield coefficient
μ: Specific growth rate
μ_max_: Maximum specific growth rate
